# Decoding Plant–Environment Interactions That Influence Crop Agronomic Traits

**DOI:** 10.1093/pcp/pcaa064

**Published:** 2020-05-11

**Authors:** Keiichi Mochida, Ryuei Nishii, Takashi Hirayama

**Affiliations:** p1 RIKEN Center for Sustainable Resource Science, Tsurumi-ku, Yokohama, Japan; p2 Kihara Institute for Biological Research, Yokohama City University, Totsuka-ku, Yokohama, Japan; p3 Graduate School of Nanobioscience, Yokohama City University, Kanazawa-ku, Yokohama, Japan; p4 Institute of Plant Science and Resources, Okayama University, Kurashiki, Japan; p5 School of Information and Data Sciences, Nagasaki University, Nagasaki, Japan

**Keywords:** Genome to phenome, Life-course approach, Multi-omics, Plant phenomics, Sensor

## Abstract

To ensure food security in the face of increasing global demand due to population growth and progressive urbanization, it will be crucial to integrate emerging technologies in multiple disciplines to accelerate overall throughput of gene discovery and crop breeding. Plant agronomic traits often appear during the plants’ later growth stages due to the cumulative effects of their lifetime interactions with the environment. Therefore, decoding plant–environment interactions by elucidating plants’ temporal physiological responses to environmental changes throughout their lifespans will facilitate the identification of genetic and environmental factors, timing and pathways that influence complex end-point agronomic traits, such as yield. Here, we discuss the expected role of the life-course approach to monitoring plant and crop health status in improving crop productivity by enhancing the understanding of plant–environment interactions. We review recent advances in analytical technologies for monitoring health status in plants based on multi-omics analyses and strategies for integrating heterogeneous datasets from multiple omics areas to identify informative factors associated with traits of interest. In addition, we showcase emerging phenomics techniques that enable the noninvasive and continuous monitoring of plant growth by various means, including three-dimensional phenotyping, plant root phenotyping, implantable/injectable sensors and affordable phenotyping devices. Finally, we present an integrated review of analytical technologies and applications for monitoring plant growth, developed across disciplines, such as plant science, data science and sensors and Internet-of-things technologies, to improve plant productivity.

## Introduction

By 2050, the human population estimated at 9.8 billion will require 25–70% more food than is currently consumed. Innovation in the current global food system is crucial to cope with this significantly increased demand and ensure that the population is fed nutritiously in a sustainable and profitable manner. In 2009, the World Economic Forum partners launched the New Vision for Agriculture as a part of a system initiative on Shaping the Future of Food Security and Agriculture, aiming to improve global food security, environmental sustainability and economic opportunity (https://www.weforum.org/projects/new-vision-for-agriculture/). Recently, this initiative presented scenarios through the analysis of the two most critical uncertainties—demand shift and market connectivity—and suggested that today’s food systems have to be overhauled to develop an efficient, sustainable, inclusive and nutritional food system for feeding future global populations (https://www.weforum.org/whitepapers/shaping-the-future-of-global-food-systems-a-scenarios-analysis).

In strengthening food security, several emerging trends pose both challenges and opportunities. Economic and population growth and rapid urbanization have been changing regional and global food consumption patterns. As shown in the 2017 Global Nutrition Report, the multiple burdens of malnutrition, including undernourishment (calorie deficiency), micronutrient (vitamin and mineral) deficiency and overnourishment [obesity and overweight, leading to diet-related noncommunicable diseases (NCD)], have been major challenges in food security and preventive health care (https://globalnutritionreport.org/reports/2017-global-nutrition-report/). Moreover, degradation of soil and water resources in the agri-food sector pose risks to crop production, which is expected to enhance the adverse effect on livelihoods and food security resulting from climate change (Pastor et al. 2019). However, emerging technologies, including plant science, data science and sensors (as part of the so-called Internet of things, or IoT), present opportunities to address these challenges through data-driven innovations in crop breeding, precision agriculture and ‘smart farming’.

To address the challenge of global food security through crop breeding, it will be imperative to integrate emerging technologies in multiple disciplines and revitalize the overall throughput of gene discovery. Therefore, in this mini-review, we aim to summarize recent advances in two major disciplines—multi-omics analysis and plant phenomics technologies—while emphasizing the potential of the life-course approach for monitoring health status in plants and crops throughout their lifespans. Specifically, we describe the recent advances in analytical technologies for monitoring health status in plants, including multi-omics-based approaches to monitor physiological status (highlighting strategies to integrate heterogeneous datasets from multiple omics areas) and phenomics techniques to noninvasively and continuously monitor plant growth (highlighting emerging technologies in spectroscopy, implantable sensors and affordable devices).

## Assessing Plant–Environment Interactions over Time

### Applying the life-course approach to plant studies

The life-course approach is an interdisciplinary study method to elucidate the relationships between earlier experiences at the beginning of life and later outcomes and well-being (Kuh et al. 2003, Halfon and Forrest 2018). In human epidemiology, the time dependency of risk factors with respect to later outcomes is a profound concept of the life-course approach, in which the longitudinal effects of such factors is often described using the concepts of timing, trajectory, transition and turning point. The approach aims to identify causal relationships between risk factors (and modifying or mediating factors) and their impacts on outcomes over time, with the causalities described using three basic conceptual models—the critical period model, the accumulation model and the pathway (chain of risk) model—and their variants (Kuh et al. 2003). The causal relationships between factors are inferred by statistical methods of causal inference, such as structural equation modeling (Warrington et al. 2019) and Bayesian inference (Madathil et al. 2018), and are often represented with a directed acyclic graph.

In human epidemiology, the life-course approach has been used to investigate biological, behavioral and psychosocial processes from gestation to adult, aiming to identify the risks and protective factors, as well as their timing and pathways, that independently or cumulatively and interactively affect chronic diseases and health outcomes in later life (Ben-Shlomo and Kuh 2002, Kuh et al. 2003, Kuh and Ben-Shlomo 2004). Specifically, life-course epidemiological studies have demonstrated that environmental exposure during earlier developmental stages can affect later pathophysiological processes, which advanced the understanding of the biological mechanisms underlying the Developmental Origins of Health and Disease approach to assess the risk factors of NCD (Hanson and Gluckman 2014, Haugen et al. 2015). With the advent of the personal genome-sequencing era (Goldfeder et al. 2017, Stark et al. 2019), coupled with digitally based transformational advances in health care and medicine, the life-course approach has attracted attention as a way to elucidate the interactions between genetic and socio-environmental factors underlying complex diseases (Halfon and Forrest 2018). Improved understanding of these interactions is expected to provide preventive and precision medicine strategies in personalized health care (Scott et al. 2019).

**Fig. 1:**
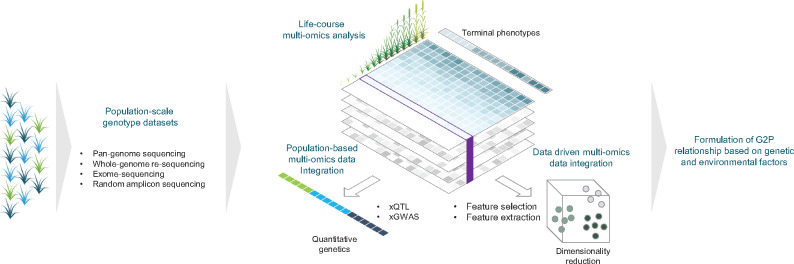
Life-course approach in crops for the formulation of genotype–phenotype relationships (G2P). Population-scale genotype datasets are obtained through genome-sequencing applications, such as pan-genome sequencing, whole-genome re-sequencing, exome sequencing and random amplicon sequencing. Physiological changes in crops are monitored using omics analyses throughout the life courses of a crop species. Multiple omics datasets are integrated by population-based approaches with quantitative genetics and data-driven approaches through strategies for dimensionality reduction.

In crop farming, agronomically important traits of plants often appear during the later growth stage and thus are significantly affected by the cumulative effects of plant–environment interactions over the growth period (Mochida et al. 2015). This naturally led us to consider applying the life-course approach in plants and crop varieties to explore the relationship between their temporal physiological response to environments across growth stages as a means to identify factors in the contexts of timing, trajectories, transitions and pathways that influence complex end-point traits such as yield, facilitating the identification of genotype-to-phenotype or genome-to-phenome (G2P) relationships in crop species (Fig. 1).

### Time-dependent description of plant growth

Time-series observations of biological phenomena are a primary approach to elucidate causal relationships between factors and later outcomes along the life-course in plants. Living organisms are open systems in which biological phenomena continuously change over time and interact with external factors (Von Bertalanffy 1950), and they are thus often described as state-transition systems, a model that is useful for understanding the causalities of biological consequences, such as development, growth, disease and adaptation. Time-series observations of plant physiological responses to environmental changes have revealed molecular mechanisms underlying fairly immediate responses to abiotic and biotic stresses (Withers and Dong 2017, Fichman and Mittler 2020), as well as longer responses to ambient environments throughout lifespans, such as seasonal adaptation (Nagano et al. 2019), stress memory and acclimation (Crisp et al. 2016, Chun et al. 2019). Moreover, recent time-series physiological studies in various plant species have demonstrated that longer physiological responses often depend on genetic variations, as well as on plant age and stage (Hara et al. 2019, Ohnishi et al. 2019). Since plants are exposed to multiple and recurring stresses, they balance resource investment through leaf-age-dependent stress-response prioritization to cope with combined stresses and to maintain growth and reproduction (Berens et al. 2019), reacting appropriately to both environmental and developmental signals to ensure survival and reproductive success. Recent studies have illustrated that these well-coordinated plant responses to the environment are actualized through cross-talk between plant hormones mediated by elaborate signaling networks (Caarls et al. 2016, Shu et al. 2018, Yang et al. 2018, Itoh et al. 2019), which are often genetically diversified through intraspecific variations (Nam et al. 2017). These findings from time-series physiological studies demonstrate that multi-omics studies can be used to explore novel relationships between molecules fluctuating in response to environmental changes across multiple omics areas.

### Emerging omics areas that facilitate understanding physiological responses in plants

Coupling with innovative analytical techniques, multi-omics studies have been popular for characterizing complex biological phenomena, and new omics areas have also emerged that facilitate the understanding of plant physiological responses. The early success of combinatorial approaches using multiple omics datasets has demonstrated the advantages of describing the states of biological phenomena based on multifaceted omics areas, as compared with single-omics-based approaches (Mochida and Shinozaki 2010, 2011). Multi-omics analysis has been applied to address biological phenomena observed in diverse plant species.

For example, the combinatorial approaches of shotgun proteomics and RNA sequencing-based transcriptomics were used to study shikonin biosynthesis in *Lithospermum erythrorhizon* (Takanashi et al. 2019). Combinatorial approaches based on genome-scale methylation profiling (methylome) and transcriptome analysis were also applied to study molecular systems underlying sex conversions in persimmon (Masuda et al. 2020), flower bud formation in apple (*Malus domestica* Borkh.) (Xing et al. 2019) and physiological responses in Arabidopsis (*Arabidopsis thaliana*) roots under zinc deficiency (Chen et al. 2018b). Emerging new omics areas coupled with innovative analytical techniques have enabled the illumination of new molecular spaces, as in lipidomics (Brügger 2014) and ionomics (Huang and Salt 2016).

Lipidomics, a focused sub-area of metabolomics that provides a comprehensive characterization of the lipids in organisms, has shed light on the metabolism and diversity of the plant lipidome (Horn and Benning 2016). In plants, lipidome profiling has facilitated the analysis not only of oil-related traits (Oenel et al. 2017) but also of physiological responses to the environment through remodeling, signaling and oscillation of membrane lipids (Nakamura 2018, Perlikowski et al. 2020). In maize (*Zea mays*), population-scale lipidome profiling combined with transcriptome analysis of a recombinant inbred line population revealed genetic factors associated with oil concentration and composition in the maize kernel (de Abreu et al. 2018), and mass spectrometry imaging-based lipid profiling illustrated the anatomical distribution of lipids, and its genetic diversity, in maize leaves (Duenas et al. 2017).

Ionomics, which focuses on the total elemental composition of organisms, has been important for elucidating the regulatory mechanisms of mineral homeostasis in plants, including uptake, transport, utilization and storage, as well as how those change in response to environmental constraints (Huang and Salt 2016). For example, ionome profiling of 19 elements in a diverse panel of maize varieties grown under different phosphorus levels and symbiotic conditions demonstrated the variety-specific effect of symbiosis with the arbuscular mycorrhizal fungus *Funneliformis mosseae* on the maize ionome (Ramirez-Flores et al. 2017). A combinatorial approach using ionome and transcriptome profiling recently illustrated plastic systems for mineral transportation in response to different soil water conditions in rice (Wang et al. 2020). In addition to organ-specific nutrient sensing, the mineral-nutrient-related long-distance signaling networks between organs have also been attracting attention as researchers work to elucidate the nutrient cross-talk that occurs during physiological responses to environmental changes in plants (Ruffel 2018).

Moreover, the development of analytical high-throughput sequencing techniques with improved affordability, throughput and multiplexing and sensitivity has provided time-, spatially- and single-cell-resolved transcriptomic and epigenomic analyses, which reveal the cellular heterogeneity and cell-type-specific states of the transcriptome and chromatin, respectively (Lee et al. 2019, Ryu et al. 2019, Torii et al. 2020). Strategies for identifying associations between omics datasets may be roughly classified into quantitative genetics-based approaches and data-driven approaches. On the one hand, quantitative genetics-based approaches have allowed us to explore the genetic association and/or linkage between genome-scale variation data and omics profiles used as a series of quantitative traits (Hasin et al. 2017). On the other hand, the data-driven approaches, which are further classified into supervised and unsupervised methods (Huang et al. 2017), have helped us identify biomarkers, molecular networks and molecular signatures that represent hallmarks of complex biological phenomena. These emergent omics areas have generated new data layers that facilitate the representation of physiological responses to environmental changes and the reconstruction of biomolecular networks across multiple omics data layers.

### Population-scale omics data for G2P modeling

Population-scale applications of multi-omics studies have provided invaluable resources to identify omics-based features that now-cast and forecast biological states, as well as genetic and environmental factors to model G2P relationships. In the medical sciences, population-scale omics data resources have been developed through coordinated projects. The Cancer Genome Atlas program (Cancer Genome Atlas Research Network 2013) is a comprehensive, coordinated project that provides an information resource comprising over 2.5 PB of genomic, epigenomic, transcriptomic and proteomic datasets and is significantly improving the understanding of cancer genetics and its application in clinical approaches. Specifically, the large-scale cancer data have enabled the development of machine-learning-based predictive models that facilitate the prediction of cancer progression (Kourou et al. 2015, Chaudhary et al. 2018), the classification of cancer subtype discovery (Gao et al. 2019) and the identification of useful biomarkers (Way et al. 2018). The Tohoku Medical Megabank Project is a large-scale project that facilitates multi-omics cohort studies aimed to advance personalized health care and precision medicine through association studies, such as metabolome and genome-wide association studies (Koshiba et al. 2018).

In plants, population-scale sequencing has been used to decipher variations in genetic codes, generating genomic resources that are useful in identifying G2P relationships. In Arabidopsis, in addition to the whole-genome re-sequencing datasets and variation map of 1,135 natural inbred lines (The 1001 Genomes Consortium 2016), the 1001 Epigenomes Project developed methylomes for 1,028 accessions and transcriptomes for 998 accessions (Kawakatsu et al. 2016). In some cereal crops, population-scale sequencing projects have provided genome-scale intraspecies variation datasets: e.g. pan-genome sequencing of 3,010 diverse Asian rice cultivars (Wang et al. 2018a), whole-genome re-sequencing of 302 soybean accessions (Zhou et al. 2015), pan-transcriptome sequencing of 503 maize accessions (Hirsch et al. 2014) and exome sequencing of 267 accessions and genotype-by-sequencing of 22,626 accessions in barley (*Hordeum vulgare*) (Russell et al. 2016, Milner et al. 2019). In crops, these genome-scale variation resources of diverse accessions facilitate genomic research and breeding through the understanding of G2P relationships. Moreover, investigations of host–microbe interaction have attracted attention to the association between microbiota and human health (Llorens-Rico and Raes 2019), as well as crop varieties and their agricultural outcomes (Toju et al. 2018). Recent longitudinal studies of host organisms and their associated microbiomes have revealed temporal shifts in human microbiomes related to human diseases, such as inflammatory bowel diseases (Zuo and Ng 2018) and Type 2 diabetes mellitus (Zhou et al. 2019), and in soil microbiomes associated with plant–pathogen interactions (Wei et al. 2019).

Population-based approaches with quantitative genetics have made it possible to explore the genetic associations and/or linkages between genome-scale variation data and omics profiles as a series of quantitative traits (Hasin et al. 2017). The Genotype-Tissue Expression project aims to develop a comprehensive resource to study tissue-specific gene expression and regulation based on datasets from nearly 1,000 people (https://gtexportal.org/home/). In plants, metabolome analyses have been widely applied to identify genetic linkages with metabolite profiles (mQTL analysis) accumulated in crops, such as tomatoes (Tohge and Fernie 2015), rice (Chen et al. 2018a) and maize (Li et al. 2019a), and they were recently used to explore the genetic association between population-wide variations and metabolite profiles (mGWAS) (Luo 2015, Fang and Luo 2019), superimposed on genetic loci associated with agronomic traits (Chen et al. 2016). The continual increases in the affordability of sequencing have accelerated the accumulation of static genome-sequencing data, as well as high-dimensional transcriptome, epigenome and microbiome data, posing challenges related to the development of strategies to extract features that well describe biological phenomena, without being defeated by the intrinsic complexity, dimensionality and modality of such datasets.

### Dimensionality reduction in high-dimensional omics datasets

Dimensionality reduction and selection of informative features from omics datasets are often critical to integrating heterogeneous and high-dimensional datasets from multiple omics areas. Dimensionality reduction, the transformation of high-dimensional data to low-dimensional space, involves critical preprocessing steps that are usually performed before model-based data mining of high-dimensional data. Since omics data usually contain a large number of variables compared to the limited number of observations or samples obtained in a standard biological experiment, it is common to encounter roadblocks in handling omics datasets: e.g. high dimensionality, which leads to assorted challenges; noisy attributes; and correlated attributes, which require more computing resources and negatively influence modeling accuracy. Therefore, to represent data with fewer features, the techniques of selection of a set of informative features (feature selection) and transformation of original features into a smaller number of new features (feature extraction) are used to identify informative features for dimensionality reduction, aiding the interpretation of high-dimensional data through visualization in ideal lower-dimensional space. Owing to the sparseness common in biological networks, such as gene regulatory networks (GRNs) (Koda et al. 2017) and microbial communities (Raman et al. 2019), statistical approaches based on the sparse estimation have been used for feature selection and for transforming features from multiple data types into fewer factors, facilitating the identification of key features, such as key regulatory genes in GRNs and biomarker candidates useful for diagnostics (Moon and Nakai 2018).

Multivariate analysis-based methods, such as partial least-squares regression, can provide efficient strategies to extract features from multi-omics data, which often contains many correlated variables, through projected latent features. For example, mixOmix is an integrated package providing a framework for multi-omics data integration for the identification of biomarkers and molecular signatures with such multivariate analysis-based methods (Rohart et al. 2017), which was used to integrate xylem transcriptome, metabolome and woody traits in Eucalyptus (Ployet et al. 2019). Moreover, the encoder–decoder architecture of convolutional neural networks, widely used for deep learning, enables the extraction of features from multiple sets of input features with encoding layers. Recently, this autonomous feature extraction has been applied to develop predictive models for clinical diagnosis, providing perspectives in precision medicine (Kalinin et al. 2018), and in systems biology in plant–microbe interaction (Mishra et al. 2019). Such feature extraction from high-dimensional multi-omics data is useful for dimensionality reduction, as well to help identify integrated features across multiple omics areas that can help generate plausible biological assumptions underlying complex traits.

### Growth and physiological monitoring and diagnostics in crops

Accurate and continuous detection of morphological and physiological changes in crops throughout their lifespans is an essential approach to assessing their genetic improvements from breeding programs and to improving management practices in farming. To enhance crop productivity, recent advances in sensor technologies, robotics and automation technologies and signal and image analytics have been widely implemented as frameworks for crop breeding and management. Plant phenomics is an interdisciplinary area aimed at understanding plant genotype–phenotype relationships, which is used to focus crop breeding strategies through the exploration of genetic associations between genome-scale genetic variations and large-scale phenotype datasets from high-throughput frameworks used to monitor plant growth (Yang et al. 2013, Tardieu et al. 2017, Araus et al. 2018). Precision agriculture utilizes a range of applications, including field mapping, crop scouting and yield monitoring, as management strategies to improve crop yield, operational efficiency and profitability in farming. Here, we review recent methodology advances and platforms for crop monitoring and diagnosis, which may offer new avenues for crop breeding and precision agriculture.

### Plant phenomics platforms

Plant phenotyping systems enable large-scale, high-throughput, noninvasive, real-time and continuous acquisition of growth and physiology data from plants throughout their lifespans. For simultaneous acquisition of spatial and temporal data from plants and the ambient environment, plant phenotyping systems usually incorporate sensors into mobility systems, such as tray conveyors, unmanned aerial vehicles (UAVs), unmanned ground vehicles and motorized gantries, along with software for communication, computing and data management (Mochida et al. 2019). Automated plant phenotyping platforms have been established that enable high-throughput and noninvasive two-dimensional (2D)-image-based trait quantification from aerial images of plants grown under controlled conditions, and these are widely used for time-lapse monitoring of plant growth to identify growth phenotypes in mutants (Arvidsson et al. 2011) and natural accessions (Feng et al. 2017) and phenotypes in response to environmental stresses (Granier et al. 2006, Dhondt et al. 2014, Clauw et al. 2015, Humplik et al. 2015). Plant phenotyping systems with automatic watering and rotation systems for individual pots provide higher spatial homogeneity, improving experimental reproducibility and allowing the precise monitoring of plant responses to soil and water conditions (Fujita et al. 2018).

### Three-dimensional plant phenotyping

Recently, three-dimensional (3D) scanning and imaging techniques have been applied in plant phenotyping, making it possible to identify and monitor geometric parameters in plant growth and traits. As 3D imaging is more robust than conventional 2D imaging with respect to occlusion due to overlapping plants and organs, plant phenotyping based on 3D imaging has recently attracted attention as a way to monitor plant architectures. As recently reviewed (Paulus 2019), 3D-imaging-based plant phenotyping has been extended to various crop species, traits and scales, with new developments including optical distance measurement techniques for 3D reconstruction, as well as improvements to sensor accuracy. Notably, 3D reconstruction methods are mainly classified into two types: active-based methods with 3D sensors for real-time depth measurements, such as light detection and ranging, structured light and time-of-flight sensors, and passive-based methods with photogrammetry for 3D modeling, such as stereovision and structure from motion. In field-scale crop phenotyping, 3D sensors have been incorporated into mobile platforms, such as mobile robots (Qiu et al. 2019) and tractors (Jiang et al. 2018, Wang et al. 2018b), and are used to phenotype geometric traits, such as plant height, above-ground biomass and growth rate. Photogrammetry-based 3D modeling is often carried out using UAV-based platforms for remote sensing and is widely used in field-scale crop phenotyping (de Castro et al. 2019, López-Granados et al. 2019). Moreover, 3D information about the physical shape of plants is combined with 2D images synchronously acquired by sensors, such as hyperspectral, multispectral, thermal and near-infrared (NIR) cameras, improving our understanding of the spatial and temporal relationship between plant morphological and physiological parameters.

### Phenotyping in plant roots

Often termed ‘the hidden half’ of plants (Eshel and Beeckman 2013, Atkinson et al. 2019), plant roots are at the frontier of plant science and crop breeding, as recent work has drawn attention to fact that their structure, anatomy, function and interactions with soil conditions greatly influence plant productivity (Bishopp and Lynch 2015, Downie et al. 2015, Ryan et al. 2016). The identification of root-related traits and their genetic control can be a promising strategy to increase agricultural yields of crops (Bray and Topp 2018). As reviewed in Atkinson et al. (2019), techniques for nondestructive plant root phenotyping have emerged based on 3D imaging techniques, such as MRI, X-ray computed tomography (CT) and positron emission tomography. Time-series data collection using these 3D imaging techniques permits spatial and temporal four-dimensional measurements, making it possible to quantify root growth (van Dusschoten et al. 2016, Jiang et al. 2019). Transparent rhizosphere imaging with transparent soil or rhizotrons (such as rhizotron plates and rhizotron tubes) allows the use of red–green–blue color and hyperspectral sensors to evaluate physiological states of plant root–microorganism interactions (Bodner et al. 2018, Ma et al. 2019). In phenotyping root system architecture in the field, a widely used approach is ‘shovelomics’, which remains a labor-intensive and destructive measurement method. Therefore, automated, nondestructive data collection methods for longitudinal monitoring of root system architectural traits from crops grown under field conditions have begun to be developed (Wasson et al. 2020), with nondestructive methods, such as ground-penetrating radar (Delgado et al. 2017) and low-cost X-ray CT (https://arpa-e.energy.gov/?q=arpa-e-programs/roots), combined with mobile frameworks providing promising techniques for automated rhizosphere monitoring.

### Plant monitoring with implantable/injectable sensors

Nanotechnology-based flexible electronic technologies have rapidly advanced and enabled the design of wearable and implantable devices for continuous, real-time, in vivo monitoring of molecular parameters and vital signs in biomedical and healthcare applications (Ling et al. 2018). Implantable/injectable sensors have also facilitated the monitoring of physiological states in plants throughout their lifespans (Giraldo et al. 2019). In the past decade, advances in nanofabrication technologies, microfluidic technologies and flexible and biocompatible electronics have, with the emergence of the IoT paradigm, enabled the fabrication of wearable and implantable sensors and their networks, which achieve noninvasive or minimally invasive, real-time, long-term and continuous health monitoring (Han et al. 2017, Koydemir and Ozcan 2018, Byun et al. 2019, Nightingale et al. 2019). Wearable and implantable/injectable medical devices have been successfully used to measure biomedical parameters (Huang et al. 2019) and have opened up new avenues for human–machine interfaces, allowing further augmentation of human abilities (Park et al. 2018). Carbon nanomaterials, such as graphene and carbon nanotubes, have been utilized as biosensors, taking advantage of their physical, chemical and electrical properties (Oren et al. 2017, Pena-Bahamonde et al. 2018). As recently reviewed in Giraldo et al. (2019), single-walled carbon nanotube-based NIR sensors embedded in plant leaves have been used to monitor signaling molecules, such as reactive oxygen species, nitric oxide (NO), calcium, glucose and ethylene (Esser et al. 2012, Giraldo et al. 2019). In addition, graphene-based wearable sensors have been employed to monitor signals associated with water transport in plants (Oren et al. 2017). Graphene-based sensors also can be integrated to wireless circuits (Park et al. 2016). Stretchable and vapor material-based sensors compatible with living leaf surfaces have enabled the longitudinal monitoring of slow, subtle physiological changes in plants throughout their growth periods (Kim et al. 2019, Zhao et al. 2019).

### Affordable plant phenotyping

Open-source frameworks may facilitate quick and cost-effective prototyping and customization of functions to develop affordable plant phenotyping systems. Recently, an international group of researchers reviewed the costs of components for plant phenotyping, including imaging devices and sensors and investment costs, and discussed the possible scenarios for affordable plant phenotyping (Reynolds et al. 2019). Small, affordable open-source single-board computers (SBCs) have allowed the development of ‘smart devices’ that integrate sensors and are incorporated into mobile platforms, playing a significant role in the emerging IoT paradigm. For example, the Raspberry Pi series, a widespread SBC platform, has been used to develop an affordable phenotyping system to monitor the growth of plants under controlled and field conditions (Dobrescu et al. 2017, Czedik-Eysenberg et al. 2018, Tovar et al. 2018). By combining Raspberry Pi-based sensors with open-source computer numerical control frameworks originally developed for gantry automation, Lien et al. (2019) created an affordable automated plant imaging system with a hyperspectral sensor for plant phenotyping. Phenotiki provides an image-based plant phenotyping platform that includes a Raspberry Pi-based device for plant image acquisition and software tools for image analyses (Minervini et al. 2017). In addition, several open-source software and hardware for plant image analyses exist: e.g. the Integrated Analysis Platform (https://sourceforge.net/projects/iapg2p/) was developed to provide an analytical pipeline of plant images (Klukas et al. 2014) implemented on the ‘PhenoBox’ platform (https://github.com/Gregor-Mendel-Institute/PhenoBox-System) (Czedik-Eysenberg et al. 2018) and the Phenotyping Hybrid Information System (http://www.phis.inra.fr/openphis/web/index.php) was proposed to manage and visualize multisource, multiscale plant phenotyping datasets (Neveu et al. 2019). Moreover, smartphone applications for point-of-care testing have burgeoned (Vashist et al. 2015, Kanakasabapathy et al. 2017) and are also used for plant disease diagnostics (Li et al. 2019b). These affordable hardware and software frameworks will promote do-it-yourself (DIY)-based phenotyping in diverse plant species, furthering our discovery of decisive developmental events, as well as of the genetic and environmental factors associated with agronomic traits in plants throughout their lifespan.

### Phenotyping of physiological responses in plants

The noninvasive and continuous monitoring of physiological responses is a major application of plant phenotyping for breeding physiological traits in crops. Photosynthesis-related traits, such as chlorophyll content, reflectance and fluorescence, have been widely used to monitor physiological states in plant species under different conditions and across genotypes (Šebela et al. 2018, van Bezouw et al. 2018, Furbank et al. 2019, Pérez-Bueno et al. 2019). Using the dynamic environmental photosynthesis imager, a phenotyping platform that enables the simultaneous measurement of photosynthetic parameters on a number of plants under dynamic or fluctuating light conditions, Cruz et al. (2016) identified ‘emergent phenotypes’ that had not been characterized under standard laboratory conditions but are observable under progressive and dynamic illumination conditions. This finding demonstrates that continuous high-throughput phenotyping under dynamically changing conditions can facilitate the identification of highly temporal phenotypes whose observation depends on environmental conditions and developmental stages. Moreover, advances in imaging techniques, including spectroscopic modalities, imaging probes and CT, have provided new avenues for plant phenotyping through space- and time-resolved imaging. For example, using the Raman spectra of plants treated with abiotic stresses, Altangerel et al. (2017) demonstrated a relationship between anthocyanin and carotenoid levels and exposure to abiotic stress. Synchrotron radiation (SR)-based applications, such as SR-Fourier transform infrared spectroscopy and SR X-rays, can be utilized to visualize the in situ distribution of biomolecules, such as organic compounds, metals and salts, as was well reviewed by Vijayan et al. (2015). Although imaging techniques based on SR require beamline facilities and are still limited in sample types and throughput, their application would potentially facilitate examination of the ultrafast kinetics of biomolecules in response to environmental stresses in plants. Radioisotopes facilitate the visualization of mineral uptake and transport, distribution and photosynthate dynamics in plants, with the real-time radioisotope imaging system allowing the acquisition of the radiation profile images in plants (Sugita et al. 2016). Although radioisotope imaging requires specific instruments combined with a fiber-optic plate with scintillator for real-time radioisotope imaging, this can be expected to illustrate physiological responses accompanying the movement of ions and photosynthates through the plant body in response to environmental changes.

## Conclusions and Prospects

In this mini-review, we have summarized recent advances in two major areas—the combination of multi-omics analysis with life-course analysis and the frameworks for plant phenotyping—that are poised to synergistically advance our understanding of plant–environment interactions. To meet the increasing global demand for food production and environmentally sustainable intensification of agriculture, interdisciplinary approaches need to be established across plant science, sensor technology, nanotechnology, data science and IoT technology to ensure sustainable agricultural production. High-throughput sequencing techniques have provided new avenues, such as Genebank-scale genomics, that will make it possible to leverage genetic variation within a crop species and plant phenotypic data to facilitate breeding programs (Milner et al. 2019). In addition to imaging-based plant phenotyping, nanotechnology-based implantable sensors will provide techniques to continuously monitor the health status of plants, which may provide novel insights into plant–environment interactions through longitudinal continuous monitoring of plant status with high spatiotemporal resolution (Giraldo et al. 2019). The availability of SBCs has accelerated the prototyping of affordable DIY sensors for plant phenotyping, and SBCs ready for deep learning may enable the use of artificial-intelligence-based edge computing to diagnose crop status based on continuously monitored biomarkers. The high-dimensional datasets produced by multi-omics and phenome analyses have posed challenges for interpretation, requiring the development of relevant strategies for dimensionality reduction. For example, by combining multiple sensors and omics profiles to describe physiological response to environmental conditions in depth, Perlikowski et al. (2020) recently demonstrated integrated features that were potentially related to a trait for drought-avoidance strategy in *Festuca arundinacea* through comprehensive time-series analytics, including analyses of root architecture, phytohormones, proteome, primary metabolome and lipidome under progressive stress conditions. During the last decade, convolutional deep neural networks have been widely used in computer vision applications, enabling the extraction of informative features from multimodal datasets, such as multi-omics data and medical images, for use in now-casting and forecasting human health status for personalized healthcare and precision medicine. This approach may further facilitate the extraction of features from data ranging from genome to phenome datasets even in crop varieties. These advances in analytical techniques will provide a framework to digitize plant–environment interaction data and monitor plant health status, thereby facilitating crop breeding, precision agriculture and smart farming to achieve global food security.

## Funding

Grant-in-Aid for Scientific Research (C) [19K11861 to K.M. and R.N.] of the Japan Society for the Promotion of Science (JSPS) and JST CREST [ JPMJCR16O4 to K.M. and T.H.].

## Disclosures

The authors have no conflicts of interest to declare.
